# Supplementary material for: Spatiotemporal regulation of the hepatocyte growth factor receptor MET activity by sorting nexins 1/2 in HCT116 colorectal cancer cells
Source: Biosci Rep. 2024 Jun 21;44(6):BSR20240182. doi: 10.1042/BSR20240182 (PMC11196213; doi:10.1042/BSR20240182)
Supplement: Supplementary Figures S1-S5 and Table S1 [file BSR-2024-0182_supp.pdf]

Title:
Spatiotemporal regulation of the hepatocyte growth factor receptor MET activity by sorting nexins 1 and 2 in HCT116 colorectal cancer cells

Authors:
Laiyen Garcia Delgado[1,3,5*], Amélie Derome[1,3,5*], Samantha Longpré[1,3], Marilyne Giroux-Dansereau[1,3], Ghenwa Basbous[2,5], Christine L. Lavoie[1,3,4,5,§], Caroline Saucier[2,4,5,§], Jean-Bernard Denault[1,3,4,5,§]

Affiliations:
[1] *Department of Pharmacology and Physiology;*
[2] *Department of Immunology and Cell Biology, Faculty of Medicine and Health Sciences;*
[3] *Pharmacology Institute of Sherbrooke (IPS);*
[4] *Centre de Recherche Clinique CHUS;*
[5] *Université de Sherbrooke's Cancer Research Institute (IRCUS), Université de Sherbrooke, 3001 12e Avenue Nord, Sherbrooke, QC J1H 5N4, CANADA*
*: These authors contributed equally.
§: These authors share senior authorship.

Supplementary Material:
Supplementary table (Table S1)
Supplementary figures (Figures S1-S5)

Table S1. Antibodies used.

Antibodies for immunoblotting			
**Antibody**	**Dilution**	**Catalog number**	**Source**
MET (D-4)	1:1000	sc-514148	Santa Cruz Biotechnology (Dallas, TX, USA)
SNX5 (F-11)	1:1000	sc-515215	Santa Cruz Biotechnology
SNX6 (D-5)	1:1000	sc-365965	Santa Cruz Biotechnology
SNX2	1:1000	Y00322-002	Immune Biosolutions (Sherbrooke, QC, Canada)
SNX1 (51)	0.25 µg/mL	611482	BD Biosciences (Franklin Lakes, NJ, USA)
PARP-1 (C2-10)	556362	1:7500	BD Biosciences
Phospho-MET (Tyr1234/1235) (D26)	1:1000	3077	Cell Signaling Biotechnology

			(Danvers, MA, USA)
Phospho-p44/42 MAPK (Erk1/2) (Thr202/Tyr204)	1:2000	4370	Cell Signaling Technology
p44/42 MAPK (Erk1/2)	1:1000	4695	Cell Signaling Technology
Phospho-AKT (Ser473)	1:500	9271	Cell Signaling Technology
AKT	1:500	9272	Cell Signaling Technology
E-cadherin (36)	0.25 µg/mL	610181	BD Biosciences
Actin (AC-40)	1:5000	A3853	Sigma-Aldrich (St-Louis, MO, USA)
α-tubulin (B-5-1-2)	1:5000	T5168	Sigma-Aldrich
Anti-mouse IgG, HRP-linked	1:5000	7076	Cell Signaling Technology
Anti-rabbit IgG, HRP-linked	1:5000	7074	Cell Signaling Technology
Anti-chicken IgG, HRP-linked	1:3000	Y00008-002	Immune Biosolutions

Antibodies for immunofluorescence			
**Antibody**	**Dilution**	**Catalog number**	**Source**
MET (L6E7)	1:1000	8741	Cell Signaling Technology
EEA1	10 µg/mL	PA1-063A	Thermo Fisher Scientific (Waltham, MA, USA)
CD71 (D7G9X)	1:100	13113	Cell Signaling Technology
LAMP1 (D2D11)	5 µg/mL	9091	Cell Signaling Technology
RAB7 (D95F2)	1:50	9367	Cell Signaling Technology
GGA3	1 µg/mL	PA5-82888	Thermo Fisher Scientific

Goat anti-Rabbit IgG (H+L) Cross-Adsorbed Secondary Antibody, Alexa Fluor™ 405	12 ng/mL	A31556	Thermo Fisher Scientific
Donkey anti-Rabbit IgG (H+L) Highly Cross-Adsorbed Secondary Antibody, Alexa Fluor™ 488	12 ng/mL	A21206	Thermo Fisher Scientific
Donkey anti-Rabbit IgG (H+L) Highly Cross-Adsorbed Secondary Antibody, Alexa Fluor™ 594	12 ng/mL	A21207	Thermo Fisher Scientific
Donkey anti-Mouse IgG (H+L) Highly Cross-Adsorbed Secondary Antibody, Alexa Fluor™ 488	12 ng/mL	A21202	Thermo Fisher Scientific
Donkey anti-Mouse IgG (H+L) Highly Cross-Adsorbed Secondary Antibody, Alexa Fluor™ 594	12 ng/mL	A21203	Thermo Fisher Scientific
Alexa Fluor™ 568 Phalloidin	1:1000	A12380	Thermo Fisher Scientific

Antibodies for cytometry			
**Antibody**	**Dilution**	**Catalog number**	**Source**
Human HGFR/c-MET Alexa Fluor® 488-conjugated	5 µL/10^6 cells	FAB3582G	R&D systems (Minneapolis, MN, USA)

Figures S1-S5

A

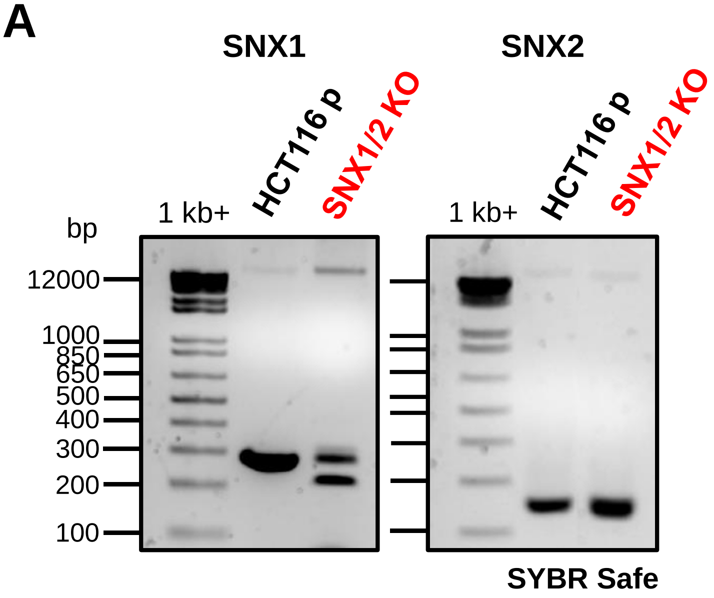

B

Cells	SNX1 sequence	SNX2 sequence
**HCT116 p**	**wt**	**wt**
**SNX1/2 KO**	**68 nt deletion**<br>**11 nt deletion**	**4 nt deletion**<br>**1 nt insertion**

Suppl. Figure 1: The knockout of *SNX1* or *SNX2* in HCT116 cells was validated by PCR and sequencing. A Genomic DNA from parental and *SNX1/2* KO cells were subjected to PCR amplification of *SNX1* or *SNX2* specific sequences (including the CRISPR/Cas9 target region), using the primer pairs SNX1-F: 5'-GCAGTGTCAGCTGATTTGTCC-3'; SNX1-R: 5'-AACAGAAGCTTACGCGGACT-3'; SNX2-F: 5'-TGATGCTAAATTGTGCATTGCC-3'; SNX2-R: 5'-TGCAGCAAAATGTGACCATGT-3', and the PCR products were next sequenced. **B** The table shows a summary of the modifications in the sequence of *SNX1* and *SNX2* resulting in the depletion of both proteins.

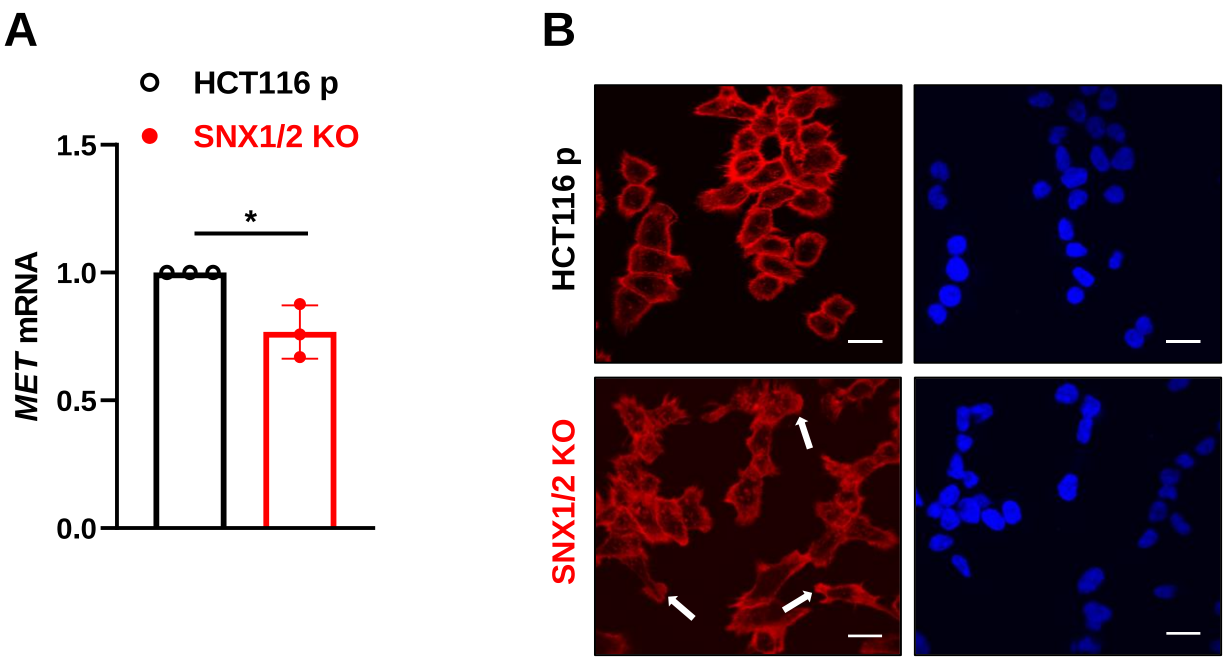

Suppl. Figure 2: The knockout of SNX1 and SNX2 decreases *MET* mRNA levels and induces actin reorganization in HCT116 cells. A The level of *MET* mRNA was determined by qRT-PCR using the primers 5'-TGGCTACACACTGGTTATCACTGG-3' and 5'-ACTGGAAATGTCTGCAGCCCAA-3'. MRPL19 (mitochondrial ribosomal protein L19), PUM1 (pumilio RNA-binding family member 1), and YWHAZ (tyrosine 3-monooxygenase/tryptophan 5-monooxygenase activation protein zeta) were used as reference genes. The bar graph represents the mean ± SD of three independent experiments, normalized to the parental cells (N=3). **B** Confocal microscopy images for polymerized actin staining (phalloidin). Nuclei were counterstained with Hoechst. Arrows indicate membrane protrusions. Scale 20 µm; 60X objective.

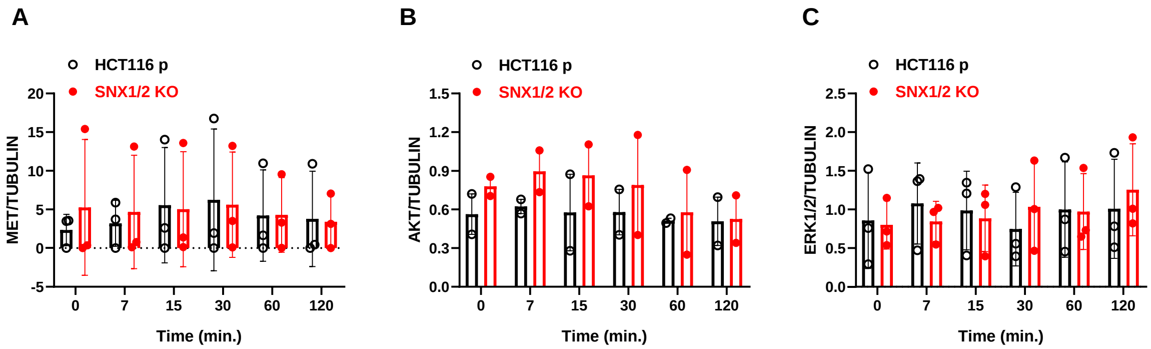

Suppl. Figure 3: The levels of total MET, ERK1/2, and AKT do not differ between parental and *SNX1/2* KO cells upon HGF stimulation. Serum-starved cells were stimulated with 50 ng/mL HGF for the indicated period. Graphs show the densitometric quantification of data from **Figure 5A**, expressed as the ratio of total **A** MET, **B** ERK1/2, and **C** AKT proteins, normalized to the loading control tubulin. Data correspond to the mean ± SD (N=3).

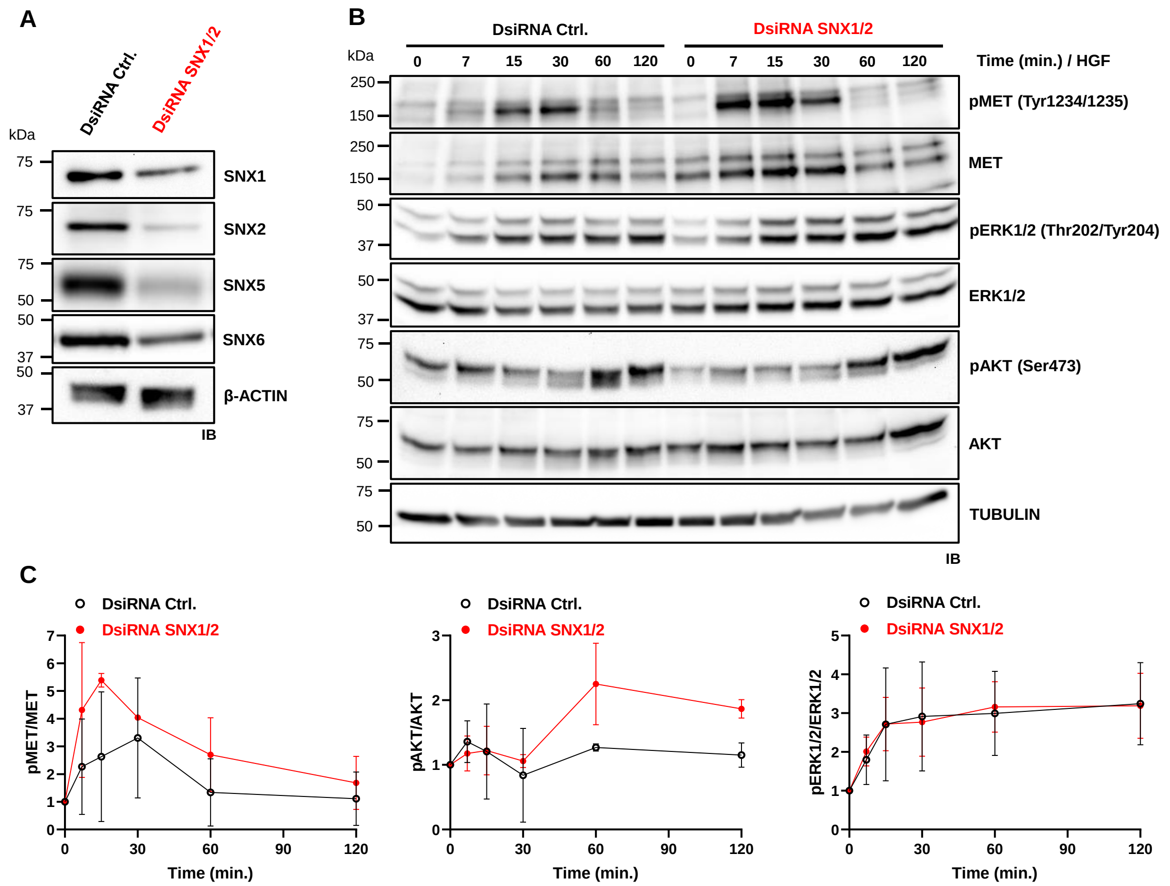

Suppl. Figure 4: The *SNX1* and *SNX2* knockdown potentiates the phosphorylation of MET and AKT upon HGF stimulation. A Validation of reduced SNX1 and SNX2 protein expression following DsiRNAs transfection, as well as of the resulting down-regulation of SNX5 and SNX6, by immunoblotting. **B** DsiRNA-transfected cells were serum-starved and stimulated with HGF (50 ng/mL) for the indicated period. Phosphorylation of MET and the downstream effectors AKT and ERK1/2 was analyzed by immunoblotting. Tubulin was used as a loading control. **C** Graphs show the densitometric quantification of data from **B** expressed as the ratio of phosphorylated protein/total protein and normalized to the unstimulated sample. Data corresponds to the mean ± SD (N=3).

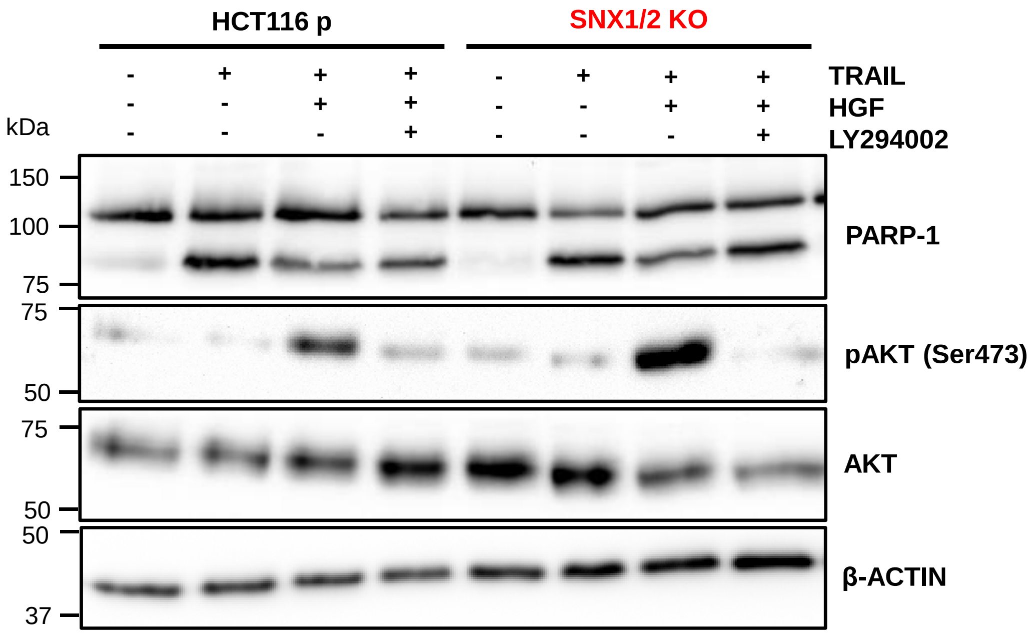

Suppl. Figure 5: The protective effect from TRAIL-induced apoptosis depends on MET-PI3K-AKT activation. Apoptosis was induced by treating cells with TRAIL (50 ng/mL). Simultaneously, cells were treated with HGF (100 ng/mL) alone or with the PI3K inhibitor LY294002 (10 µM). The cleavage of PARP-1 was determined by immunoblotting; actin was used as a loading control.
